# Egyptian evidence-based consensus on clinical practice recommendations for the management of Kawasaki disease

**DOI:** 10.1186/s43166-023-00180-2

**Published:** 2023-04-10

**Authors:** Yasser El Miedany, Samia Salah, Hala M. Lotfy, Mohammed Hassan Abu-Zaid, Sally S. Mohamed, Sheren Esam Maher, Maha El Gaafary, Hala Abdulhady, Yomna Farag, Mervat Eissa, Ahmed Radwan, Basma M. Medhat, Dalia M. E. El Mikkawy, Waleed A. Hassan, Doaa Mosad Mosa, Ghada El Deriny, Mohamed Mortada, Naglaa S. Osman, Nermeen Ahmed Fouad, Youmna Ahmed Amer, Samah Ismail Nasef, Hend Abushady, Salwa Galal, Eiman Abd El-Latif, Dina Maria, Ahmed H. Shabana, Samar AbdAlhamed Tabra

**Affiliations:** 1 Canterbury Christ Church University, Kent, England; 2 King’s College London, London, England; 3 Pediatric Rheumatology, Cairo University, Cairo, Egypt; 4 Rheumatology and Rehabilitation, Tanta University, Tanta, Egypt; 5 Rheumatology and Rehabilitation, Cairo University, Cairo, Egypt; 6 Pediatric Rheumatology, Minia University, Minia, Egypt; 7 Community Medicine and Public Health, Ain Shams University, Cairo, Egypt; 8 Rheumatology and Rehabilitation, Ain Shams University, Cairo, Egypt; 9 Rheumatology and Rehabilitation, Sohag University, Sohag, Egypt; 10 Rheumatology and Rehabilitation, Benha University, Benha, Egypt; 11 Rheumatology, Faculty of Medicine, Mansoura University, Mansoura, Egypt; 12 Pediatrics, Alexandria University, Alexandria, Egypt; 13 Rheumatology and Rehabilitation, Zagazig University, Zagazig, Egypt; 14 Pediatrics, Assiut University, Assiut, Egypt; 15 Rheumatology and Rehabilitation, Fayoum University, Fayoum, Egypt; 16 Rheumatology and Rehabilitation, Suez Canal University, Ismailia, Egypt; 17 Ophthalmology, Alexandria University, Alexandria, Egypt; 18 Cardiology, Tanta University, Tanta, Egypt; 19 Pediatric Cardiology, Tanta University, Tanta, Egypt

**Keywords:** Recommendations, KD, Kawasaki, Vasculitis

## Abstract

**Background:**

Kawasaki disease (KD) is an acute, self-limited febrile disease of unidentified cause that mostly affects children less than 5 years of age. This work aimed to provide an appropriate Egyptian evidence-based consensus on clinical practice recommendations for the management of Kawasaki disease. The main objective of this study, which employed the Delphi method, was to reach a consensus among experts on a treat-to-target management approach for KD.

**Results:**

The expert panel was confined to an online survey (*n*=26), and all the expert completed the three rounds. At the conclusion of round 3, a total of 17 recommendation items were gathered, which were divided into two sections. The range of respondents (ranks 7–9) who agreed with the recommendations was 92.3 to 100%. All 17 clinical standards identified by the scientific committee were written in the same way. There have been algorithms proposed for managing various KD conditions.

**Conclusion:**

The developed evidence-based consensus recommendations for the diagnosis and management of KD represent an up-to-date document that focuses on clinical management questions which are generally posed to health care professionals involved in the management of KD. This guideline was developed considering experience with and availability of treatment and diagnostic options in Egypt.

## Background

Kawasaki disease (KD) continues to be an area of evolving understanding in clinical treatment. Since its first description by Dr. Tomisaku Kawasaki, based on the classical clinical symptoms, Kawasaki disease has been recognized as one of the two most common systemic vasculitis in childhood (the other one is IgA vasculitis [Henoch-Schönlein]) [1]. KD is an acute, self-limited febrile disease of unidentified cause that mostly affects children less than 5 years of age. KD is a medium vessel vasculitis which prior to the identification as a distinct illness, children with fulminant forms of the disease used to be identified as “infantile polyarteritis nodosa” [2]. Although inflammation occurs throughout the body, the coronary artery is the commonest vessel to be affected and their involvement can trigger severe coronary artery aneurysms (CAA). This makes those children living with the disease a high-risk cohort of patients.

In the absence of pathognomonic tests, the diagnosis of KD remains a challenge. In addition to the absence of diagnostic tests, the diagnostic clinical criteria may develop sequentially, rather than simultaneously, which may cause further delay in making the diagnosis. Also, a variability in patient presentation has been reported, as many symptoms such as arthritis, gastrointestinal, CNS, or liver manifestations as well as uveitis may accompany the principal criteria. Furthermore, children presenting with incomplete KD never develop all the classic criteria. Several Egyptian healthcare experts have the misconception that KD is not frequent in Egypt [3].

Timely initiation of management for KD has proven its efficacy in securing favorable long-term diagnosis. Untreated KD may lead to the development of coronary artery aneurysms which may cause sudden cardiac death in children. Furthermore, as young persons with post-COVID-19 multisystem inflammatory syndrome in children (MIS-C) may present with Kawasaki-like disease, and because its management is derived from KD therapeutic options thus far, considerable attention has been paid to KD following the COVID era [3]. Given the growing number of available options to treat systemic vasculitis, there have been several reports discussing variable presentations of the disease, whether complete, incomplete, or atypical KD, in Egypt [4–7]; it was important to set national management recommendations for KD to ensure capturing and treating the disease during the effective window of therapy national guidelines for specific diseases should be based on the most recent research available at the time of release and in the context of the population’s local environment. The Egyptian College of Paediatric Rheumatology has taken this initiative and provide an up to date evidence-based consensus for the management of KD. This is in concordance with the nationwide universal health coverage approach which has been launched in Egypt in 2020. Setting up such guidelines for management of patients is vital to the process. Although intended to help Egyptian children with KD; yet, we hope it will be a beneficial simple guidance for diagnosis and management of KD all over the world.

## Methods

### Design

The development of the evidence-based consensus treatment recommendations for KD using a multistep process strategy. The study’s design was developed in accordance with the CEG guideline development process methodology, which relies on existing scientific data and clinical experience to reach consensus. The publication complied with the criteria for reporting systematic reviews and meta-analyses on preferred reporting items for such studies [8].

### Development stages

#### Core team

Three experts with proven KD management experience made up the team. The core team managed and oversaw the team’s efforts, helped define the project’s parameters and the original Patient/Population, Intervention, Comparison, and Outcomes (PICO) clinical questions, and came to an agreement on the guidelines’ most important inquiries. The core team pre-identified outcomes for each PICO question that were essential for the systematic literature evaluation. The team also proposed the panel of experts and prepared the manuscript.

#### Key questions used to develop the guideline

The target population, the intervention, the investigation, the comparison(s) employed, and the outcomes used to quantify efficacy, effectiveness, or risk were all defined in this guideline. The stages for collecting the evidence to address the clinical questions were as follows: formulation of the clinical questions, question structuring, search for the evidence, critical evaluation and selection of the evidence, presentation of the findings, and recommendations. These inquiries, which are presented in Table 1, formed the basis for the systematic literature review and, ultimately, the clinical care standards.

**Table 1:** Key questions used to develop the guideline

1- Who are the targeted population? 2- What is the definition of typical KD, incomplete KD, atypical KD, and acute phase of KD? 3- What are the features of high-risk disease/severe disease? 4- What are the predictors for the development of coronary artery aneurysms and risk factors for IVIG resistance? 5- What is the definition of refractory/resistant KD? 6- How should KD patients assess and monitored and what is the frequency of monitoring KD? 7- What is the initial treatment of acute KD? 8- What is the initial treatment for patients with acute KD who are at high risk of IVIG resistance or developing coronary artery aneurysms? 9- What is the treatment for patients with acute KD resistant to treatment (refractory KD)? 10- What is the treatment for patients with acute KD who have arthritis resistance after IVIG treatment and who do not have coronary artery aneurysms KD? 11- What is the management of the ophthalmologic manifestation of KD? 12- What is the treatment for patients with incomplete KD? 13- What is the maintenance treatment in a patient with KD after an acute attack (treatment of cardiac complication)? 14- What is the prevention and treatment of thrombosis in patients with coronary aneurysms? 15- What is the treatment for patients with acute KD and complicated with macrophage activation syndrome? 16- What about vaccinations in a child with KD? 17- What is the long-term follow-up?	

*KD *Kawasaki disease

#### Literature review team

The literature evaluation was carried out with the assistance of a methodology expert under the direction of an experienced literature review expert and based on the particular research questions established to concentrate on the management of KD. Using the PubMed/MEDLINE, EMBASE, and Cochrane databases, a systematic literature search was conducted to gather the necessary evidence-based background knowledge for deliberations. The experts in responsible of the literature review revised the data after it had been abstracted, looked over published recommendations, and rated the quality of the evidence [9, 10]. They then provided a thorough list of recommendations for the management of KD based on the research evidence that was currently available and their own clinical expertise. The Oxford Centre for Evidence-based Medicine (CEBM) approach was used to establish the level of evidence for each section (Table 2) [10].

**Table 2:** Levels of evidence

Level of evidence	
1	Systematic review of all relevant randomized clinical trials or n-of-1 trials
2	Randomized trial or observational study with dramatic effect
3	Non-randomized controlled cohort/follow-up study (observational)
4	Case series, case-control study, or historically controlled study
5	Mechanism-based reasoning (expert opinion, based on physiology, animal, or laboratory studies)
Grades of recommendation	
A	Consistent level 1 studies
B	Consistent level 2 or 3 studies, or extrapolations from level 1 studies
C	Level 4 studies, or extrapolations from level 2 or 3 studies
D	Level 5 evidence or troubling, inconsistent, or inconclusive studies of any level

#### Data sources and search strategies

The search approach was designed to find all studies involving children living with KD as the study population. The literature search was done using the PICO questions (Table 1). To find randomised clinical trials testing the effectiveness of KD care as well as quality improvement outcomes/approaches, literature search methodologies were used.

The chosen keywords were determined by the various PICO element combinations. PubMed, Cochrane Library, and Embase database searches will take place on January 12, 2022, and January 25, 2022, respectively. Updated on April 4th, 2022, was the search. Electronic duplicate screening of literature search results was done. Reviewing the reference lists of studies found through database search tactics that satisfied the inclusion criteria allowed for the retrieval of additional pertinent studies.

### Study selection

Applying inclusion and exclusion criteria to the literature retrieved using the search strategies allowed for the selection of pertinent research.

#### Inclusion criteria

Systematic reviews, randomized controlled trials (RCTs), uncontrolled trials, observational studies including cohort, case control, and cross-sectional studies, as well as articles where an economic evaluation was done, were all included in the list of articles. Trials were considered eligible if they involved children, regardless of gender, from any medical environment receiving any therapy who had been diagnosed with KD. The classification evidence and suggestions utilised in the included research should be identified. The formal procedure for making recommendations (Delphi exercise, panel conference) is also described.

#### Exclusion criteria

Editorials, commentary, abstracts from conferences, narrative/personal reviews without supporting data, and articles without an English translation were excluded.

#### Expert panel

There were 26 members nominated by the core leadership group. A minimum of 8 years of professional experience in the field of rheumatology, management of inflammatory arthritis, specifically KD, and active engagement in rheumatic disease research were requirements for their selection.

The expert panel, which included ophthalmologists, hematologists, pediatric cardiologists, and rheumatologists, provided professional knowledge to help with discussions of the PICO questions and literature review’s conclusions. PICO questions were turned into recommendation statements and forwarded with the evidence report to the expert panel for voting.

#### Target audience

The aim of the recommendation is to help medical professionals, such as rheumatologists, paediatricians with a focus on pediatric rheumatology, cardiologists, hematologists, dermatologists, and ophthalmologists, who treat and manage patients with KD. The recommendation should serve as a useful tool for patients and those in charge of commissioning treatment for KD patients under the National Health Service.

### Developing the clinical care standard framework

To promote consistent identification of guideline components, a structured template was developed based on the responses to the structured key questions and the literature review. The format in which recommendations and information will be supplied and extracted for each guideline component has been determined.

#### Delphi process

The Delphi method is a structured approach that is frequently used to collect crucial data on a certain subject. It is predicated on the fundamental tenet that group projections are typically more accurate than individual ones. In order to create consensus forecasts from a group of experts in an organised, iterative manner, the Delphi method was developed. Its methodology is based on a number of “rounds” of questions sent to experts. The following phases are typically covered by the Delphi method: (1) A group of experts is put together. (2) The experts are given forecasting assignments and difficulties. (3) The experts provide early predictions and explanations. These are gathered and summarised in order to offer comments. (4) The experts receive input, which they use to review their projections. Up until a suitable degree of agreement is obtained, this phase may be repeated. (5) The expert forecasts are combined to create the final projections. The participants in this method are anonymous, and the feedback is carefully regulated [11–13].

#### Consensus process

To reach consensus on the T2T (treat to target) strategy in KD, three Delphi rounds were conducted. The organized Delphi process makes sure that all participant opinions are taken into account. Online surveys were used to carry out the Delphi process. Seventeen items from the T2T strategy of KD were included in the first round of the electronic questionnaire.

### Voting process

Voting took place in three rounds of precisely timed live online delivery. All task force members received invitations to vote and advance notice of the start and end times of each round. Anonymous votes were gathered and processed, and special access links were distributed. During the voting process, comments on rephrasing, potential ambiguity, and unidentified overlaps was obtained in relation to each statement. Voting on the statements was only permitted for task force participants.

### Rating

Each statement was given a score ranging from 1 to 9, with 1 denoting “total disagreement” and 9 denoting “full agreement.” The numbers 1–3, 4–6, and 7–9, respectively, are used to denote disagreement, doubt, and agreement. Voting was not obliged on any statements, and participants were encouraged to abstain if they believed a statement to be outside of their area of expertise. Voting with “uncertainty” expresses “discomfort about the accuracy of the recommendation.” All statements were open to the entry of comments, which the scientific committee assessed following each vote session. Members were urged to comment throughout every round of voting, especially whenever there was a disagreement. With the use of this, the panel was able to determine a case of misinterpretation of a remark and invalidate the vote on it.

### Interpreting the recommendations: definition of consensus

Before data analysis, a definition of consensus was defined. It was determined that agreement (scoring 7–9) or disagreement (scores 1–3), which would then become a recommendation in this guideline, would be attained by at least 80% of participants [11–13]. If a statement received a “poor” degree of agreement or a mean vote that was less than 3, it was retired. In view of the comments, statements whose rating fell within the (4–6) range of the uncertainty score were amended. When all votes on a proposal fell into the agreement bracket after the second round of voting, the levels of agreement on each statement of recommendation were considered to be “high” (7-9) [13, 14].

### Chronogram of Delphi rounds

The first round was held from June 2 through June 6, 2022 (5 days). In light of the remarks, the elements on which respondents could not agree in this first round were amended and included in the second round. One month after the first round, on July 6, 2022, the second round took place and lasted for 5 days (6th–10th Jul 2022). The third round then began on August 7th, 2022, and lasted for 5 days (7th–11th Aug 2022).

#### Ethical aspects

In compliance with the Helsinki declaration, this study was carried out. The local ethics commission, Tanta University’s ethical board, and its ethical approval code, 34842/8/21, accepted the Clinical, Evidence-based, Guidelines (CEG) project procedure. Since there were no human participants in this study, Human Studies Committee permission was not necessary. According to national standards, written ethics permission from the experts involved in this work was not deemed essential.

## Result

### Literature research and evidence selection

By using a search strategy, we identified 2530 possibly relevant studies during the research selection phase. 2375 were dismissed due to duplicates or following title and abstract screening (studies did not examine population or intervention of interest, did not match study design of interest, or did not report outcome measures of interest). One hundred fifty-five studies that were pertinent were therefore included for the entire article review. One hundred thirty-three studies were excluded because the citations did not offer proof that matched a PICO. Twenty-two studies were therefore included in this work.

### Expert panel characteristics

The Delphi form was sent to the expert panel (*n*=26), who participated in the three rounds. The experts were 22 adult and pediatric rheumatologists, 1 pediatric cardiologist, 1 cardiologist, 1 ophthalmologist, and 1 statistician. Respondents came from various governorates and medical centers all around Egypt: Cairo University (26.9%), Ain Shams University (11.5%), Tanta University (15.4%), Benha University (3.8%), Alexandria University (7.7%), Suez Canal University (3.8%), Zagazig University (7.7%), Minia University (3.8%), Mansoura University (3.8%), Fayoum University (3.8%), Assiut University (3.8%), Sohag University (3.8%), and the UK (3.8%).

#### Delphi round 1

Round 1 had a 100% response rate (26/26). On all of the clinical key questions, an agreement was attained on 100% of the topics, meaning that 75% of respondents strongly agreed or agreed. Concerning the phrasing of some of the recommendations, there were 17 key questions and comments. Key points, disease monitoring, and treatment targets received more comments (excluding small editing suggestions). There was no diversity of opinion in round 1.

#### Delphi round 2

Round 2’s response rate was 100% (26/26) overall. A high-rank recommendation (rank 7–9) was given to between 88.5 and 100% of applicants. There was consensus (i.e., 75% of respondents strongly agreed or agreed) on all clinical standards. The remarks’ phrasing was agreed upon by all stakeholders (>80% agreement).

#### Delphi round 3

Round 3’s response rate was 100% (26/26) overall. A high-rank recommendation (rank 7–9) was given to between 92.3 and 100% of applicants. The overarching principles and recommended statements are shown in Table 3.

**Table 3:** Summary of recommendations

Standard	Statement	Mean score of agreement rate± SD	% of Agreement	Level of agreement
**Overarching principle**	KD needs a multidisciplinary team consisting of pediatric rheumatologist or rheumatologist with a special interest in pediatric rheumatology, a pediatric cardiologist, a cardiologist who is interested in pediatric rheumatology and may need other specialists such as an ophthalmologist, pediatric, hematology, etc. The child presented with KD, general pediatricians should consider the diagnosis of KD and early referral to a specialist if the child has persistent fever and 2 or more of KD characteristic symptoms (rash, red eye … ..etc.)	8.31±1.9	92.3	H
**Who are the targeted population?**	The guideline targets the management of children diagnosed with Kawasaki disease either typical or incomplete by American Heart Association diagnostic criteria. Our target physicians are pediatric rheumatologists, adult rheumatologists who are interested in pediatric rheumatology, pediatric cardiologist, a cardiologist who is interested in pediatric rheumatology, ophthalmologist, regulatory bodies, health-related organizations, and interested patients’ groups/laypersons	8.19±1.79	92.3	H
**What is the definition of typical KD, incomplete KD, atypical KD, and acute phase of KD?**	**Typical KD:** Fever lasting at least 5 days without any other explanation with at least 4 of the 5 following principal clinical findings: (1) bilateral bulbar conjunctival injection without exudate, (2) erythema and cracking of lips, strawberry tongue, and/or erythema of oral and pharyngeal mucosa, (3) erythema and edema of the hands or feet (acute phase), and/or periungual desquamation (subacute phase), (4) maculopapular, diffuse erythroderma, or erythema multiforme–like rash, or (5) cervical lymphadenopathy (at least 1 lymph node >1.5 cm in diameter), usually unilaterally. The diagnosis may be made with only 4 days of fever if ≥4 principal clinical features are present or if CAA (*Z*-score >2.5) or coronary dilatation (*Z*-score >2, but <2.5) is present. **Incomplete KD:** unexplained prolonged fever in an infant (≥ 7 days) or child (≥ 5 days) with less than 4 of the principal clinical findings of KD, and compatible laboratory markers (elevated ESR/CRP level, with 3 or more of the following laboratory findings: anemia, thrombocytosis, leucocytosis, hypoalbuminemia, Increased leukocytes in the urine sediment of an infant 10 < white cell count/hpf and elevated transaminase levels) or echocardiographic findings (coronary artery dilatation). **Atypical KD:** occurs in patients presenting a typical fever not otherwise explained, lasting for ≥5 days, and signs or symptoms that differ from the main KD clinical characteristics (i.e., meningeal inflammation, seizures, facial paralysis, acute abdomen, acute pancreatitis, cholestatic jaundice, arthritis, renal injury, pneumonia, etc.), with or without coronary artery aneurysm. **Acute KD**: Initial febrile phase of KD.	8.65±0.62	100	H
**What are features of high-risk disease/severe disease?**	**HIGH RISK**. At least one of the following: **(LOE:5D)** • Age <12 months • C-reactive protein higher than 200 mg/l • Albumin ≤ 2.5 g/dL • Hb at least 2 g/dL below the lower limit of normal for age • Liver dysfunction: AST or ALT ≥ 2x upper limit of normal and/or direct bilirubin > 1 mg/dL • overt coronary artery aneurysms • macrophage activation syndrome or septic shock • Kobayashi score ≥ 4 **Severe disease** • Shock • Age < 12 months • Presence of coronary or peripheral aneurysms or other cardiovascular abnormalities. • haemophagocytic lymphohistiocytosis	8.88±0.43	100	H
**What are the predictors for the development of coronary artery aneurysms and risk factors for IVIG resistance?**	**Predictors for the development of coronary artery aneurysms: (LOE:4C)** • a *Z*-score in the left anterior descending or right coronary artery of ≥2 • age <6 months • Asian race • a C-reactive protein level of ≥13 mg/dl • uveitis **Predictors for nonresponse to IVIG: (LOE:3C):** there is no widely accepted grading system for predicting IVIG non-responsiveness, and for the Caucasian population, there is no validated risk score yet but **Kobayashi score (≥4 points of the following) can be used** but may not reliably exclude IVIG resistance if “negative” (score <4) • Na ≤133mmol/L • AST ≥100IU/L • Day of starting treatment or diagnosis: Day 4 of illness or earlier • Neutrophils ≥80% • CRP ≥10mg/dL • Platelets ≤300,000/μL • Age (months) ≤12 months	8.76±0.51	100	H
**What is the definition of refractory/resistant KD?**	Failure to respond to IVIG therapy and consistent with recrudescent fever 36–48 h after IVIG infusion **(LOE:1A)**	8.61±0.85	92.3	H
**How should KD patients assess and monitored and what is the frequency of monitoring KD?**	• **KD should be differentiated from** Infectious diseases such as {viral (rubella, adenovirus, enterovirus, cytomegalovirus, Epstein-Barr virus, parvovirus B19, human herpes virus, scarlet fever, Mycoplasma pneumonia infection, toxic shock syndrome, staphylococcal scalded skin syndrome, bartonellosis, Rocky Mountain spotted fever, leptospirosis} and non-infectious diseases such as juvenile idiopathic arthritis, drug hypersensitivity reaction, Stevens-Johnson syndrome, polyarteritis nodosa, autoinflammatory syndromes, Multisystem Inflammatory Syndrome in Children (MIS-C), sarcoidosis and acrodynia.} The identification of viral or bacterial agents cannot exclude KD diagnosis **(LOE:3C).** • Laboratory tests are nonspecific for KD, but it can support the diagnosis in combination with classic features especially in patients with suggestive clinical features of KD **(LOE:5D).** • Infant aged 6 months or younger with fever for at least 7 days and no obvious cause should have a laboratory evaluation even if no symptoms of Kawasaki disease are evident; if signs of inflammation are seen, echocardiography should be done. I. **Laboratory test should be done** CBC, ESR, CRP, liver function test, serum Na, renal function, ferritin, and urinalysis **(LOE: 3C)** Other tests such as cerebrospinal fluid are needed only if the patient presents with signs of meningeal irritation and to rule out infectious meningitis and synovial fluid analysis if the patient presented with active inflammatory synovitis with effusion **(LOE: 5D).** II. **ECG should be performed** at baseline, as soon as the diagnosis is suspected **(LOE: 1A).** III. **Echocardiography** • Bidimensional and color-Doppler echocardiography and/or associated techniques (tissue Doppler imaging, 3D echocardiography imaging) are crucial for performing cardiac assessment in patients with KD at baseline, as soon as the diagnosis is suspected, as they are non-invasive repeatable investigations, characterized by both high sensitivity and high specificity and should be performed by a pediatric cardiologist or cardiologist with experience in the pediatric age. • Echo detects the size of the aneurysm (by *Z* score), its location, and the morphology of the aneurysm, detect the presence/absence of thrombi, and assess myocardial involvement, ventricular systolic, and diastolic function, LV wall motion, valvular regurgitation, and pericardial effusion. • Timing of echo a. In the uncomplicated cases: In all patients with a diagnosis of KD echocardiogram must be done, which should also be repeated 2, 6, and 8 weeks following the onset of the disease because CAA can be identified in the weeks that follow the diagnosis **(LOE: 1A).** b. Persistently febrile non-responders patients who have coronary artery aneurysm, decreased left ventricular function, mild-to-moderate mitral regurgitation, or severe pericardial effusion need to have echocardiograms more frequently (at least weekly) until resolution of fever, normalization of symptoms and decrease in CPR levels **(LOE:2B)**. IV. Cardiovascular CT scan: routine use is not indicated, ideally with a dual-source CT (DSCT) scanner, should be used in patients with KD to **(LOE:4C)** – confirm CAA – assess other aneurysms, both central and peripheral, throughout the entire body – detect middle-distal CAA (not typically seen at routine echocardiograms) – more precisely define the caliber and morphology of CAA – detect coronary artery thrombosis, calcification, or occlusions – detect myocardial ischemia V. Cardiovascular MR angiography: *routine use is not indicated. It* should be used in patients over 8 years with KD to **(LOE:4C)** -confirm CAA. - identify other aneurysmal dilations, either central or peripheral, in the vascular system. - assess ventricular systolic function. - detect myocardial ischemia	8.57±0.94	96.2	H
**What is the initial treatment of acute KD?**	I. Patients with complete KD criteria should be treated with high-dose IVIG (2 g/kg given as a single intravenous infusion) over 10–12 h **(LOE:1A)** • Time of administration of IVIG 1. Within 10 days of illness onset but as soon as possible after diagnosis **(LOE:1A)**. • If a child presents after the 10th day of illness (i.e., if the diagnosis was missed earlier) and exhibits either a persistent fever without other cause or coronary artery abnormalities along with ongoing systemic inflammation, as shown by an elevated ESR or CRP (CRP >3.0 mg/dL), it is reasonable to administer IVIG **(LOE: 4C)**. • Patients should be closely monitored for adverse reactions during IVIG infusion, including aseptic meningitis, Coombs-positive hemolytic anemia, and generalized infusion reactions. • Since IVIG therapy increases the ESR, it shouldn't be used to evaluate the response to IVIG. IVIG resistance should not be determined from a persistently high ESR alone. II. Moderate dose of aspirin (ASA) (30–50 mg/kg/d) must be administered until 48 h after the disappearance of fever **(LOE:1A)**. • After the child has become afebrile, aspirin should be reduced to 3–5mg/kg as a single daily dose for its antithrombotic effect **(LOE:1A)**. • In patients without CAA, low-dose aspirin is continued for approximately 6–8 weeks after KD onset. In children who develop CAA, low–dose aspirin therapy should be continued long-term, at least until the aneurysms resolve and the patient should be referred to a pediatric cardiologist for long-term follow-up **(LOE:3C)**.	8.61±0.98	96.2	H
**What is the initial treatment for patients with acute KD who are at high risk of IVIG resistance or developing coronary artery aneurysms?**	• Patients with acute KD who are at high risk of IVIG resistance or developing coronary artery aneurysms should receive initial therapy with IVIG + low-dose aspirin (3–5 mg/kg/day) + corticosteroid single intravenous pulse of methylprednisolone (10–30 mg/kg/day) **(LOE:1A)**. • Patients with acute KD who are at high risk of IVIG resistance or developing coronary artery aneurysms and glucocorticoids are contraindicated should receive initial therapy with IVIG and nonglucocorticoid immunomodulatory therapy such as infliximab, anakinra, or cyclosporine **(LOE:2B)**. • If treatment fails, additional IVIG infusion, low-dose aspirin (3–5 mg/kg/day), and three pulses of intravenous methylprednisolone (30 mg/kg/day) should be administered, followed by prednisone (2 mg/kg/day) (gradually tapered) **(LOE:3B)**.	8.88±0.43	100	H
**What is the treatment for patients with acute KD resistant to treatment (refractory KD)?**	A. Additional IVIG infusions, intravenous methylprednisolone pulses, infliximab, cyclosporine A, methotrexate, plasmapheresis, and ulinastatin are second-line treatment options for refractory KD **(LOE:2B)**. B. A second dose of IVIG (2 g/kg) is conditionally recommended in patients with **acute KD resistant** to the first IVIG infusion **(LOE:3B)**. C. Administration of high-dose pulse steroids (usually methylprednisolone 10–30 mg/kg intravenously for 3 days, followed by prednisone: 2 mg/kg/day, then gradually tapered)) may be taken into account as a potential alternative for a second IVIG infusion or for the retreatment of KD patients who have experienced recurrent or recrudescent fever after further IVIG **(LOE:3B)**. D. Infliximab [a single intravenous dose of 5 mg/kg of body weight] for IVIG- and corticosteroid-resistant KD patients may be considered **(LOE:2B)**. E. In patients with refractory KD who have not responded to the second IVIG infusion, infliximab, or corticosteroids, cyclosporine (IV 3 mg/kg/day split q12h, PO 4-8 mg/kg/day divided q12h) may be considered. Cyclosporine should be continued until the patient is afebrile and clinically improving with CRP less than 12 mg/dL or after 2 weeks of therapy. The dose should then be reduced by 10% every 3 days until it reaches 1 mg/kg/day **(LOE:4C)**. F. IL-1 blockade with anakinra (daily dose of 4–8 mg/kg of body weight for an overall period of 15 days) is highly promising in treating severe multi-refractory patients with KD, with potential benefits also on the cardiovascular complications **(LOE:4C)**. G. Canakinumab can be used off-label as a single subcutaneous injection of 4 mg/kg for a body weight ≤ 40 kg in KD patients who are resistant to IVIG **(LOE:4C)**. H. In resistant acute KD, plasma exchange and cytotoxic medications like cyclophosphamide have been used. However, usage of these medications should be restricted to patients for whom other treatments have failed due to the limited data and risks associated with them **(LOE:4C)**. I. Ulinastatin [5000 U/kg for 3–6 times per day (maximum dose: 50,000 U] has been proposed as useful in IVIG–refractory patients **(LOE:3C)**.	8.61±0.94	96.2	H
**What is the treatment of patient with acute KD who have arthritis resistant after IVIG treatment and who do not have coronary artery aneurysms KD?**	• For patients without coronary artery aneurysms who do not need to take aspirin on a long-term basis and who have arthritis that needs additional treatment, aspirin can be temporarily withheld and a brief course of NSAIDs (usually lasting 3–4 weeks) can be taken as needed **(LOE:5D)**. • Acetaminophen, a short course of glucocorticoids, or nonsystemic NSAID pain management options (e.g., a topical NSAID) can be given if long-term aspirin use is necessary owing to coronary artery aneurysms. If prolonged use of systemic NSAIDs is necessary (i.e., for more than three weeks), a pediatric hematologist or cardiologist should be consulted, especially in a patient with coronary artery aneurysms **(LOE:5D)**.	8.69±0.88	96.2	H
**What is the management of opthalmological manifestation of KD?**	• Involvement of the eye in Kawasaki disease includes conjunctival injection, keratitis, and uveitis, and it can cause blindness if neglected. • Costicosteroid eye drops are essential for the treatment of acute anterior uveitis in Kawasaki disease **(LOE:4C)**.	8.69±0.88	96.2	H
**What is the treatment for patients with incomplete KD?**	For patients with incomplete KD (who meet the criteria for incomplete KD according to the AHA guidelines). It is strongly recommended to start IVIG as soon as the diagnosis is made over delaying therapy until day 10 or later **(LOE:1A)**.	8.88±0.43	100	H
**What is the maintenance treatment in patient with KD after acute attack (treatment of cardiac complication)?**	• Patients with KD who have medium-sized aneurysms (5 mm and 7 mm or if *Z* scores 7 and 10) or those who have multiple and complex aneurysms require antiplatelet prophylaxis based on low-dose ASA (3–5 mg/kg/day) combined with clopidogrel (0.2 mg/kg/day in patients aged < 24 months or 1 mg/kg/day if age ≥ 25 months, max 75 mg/day) in a single dose **(LOE:4C)**. • KD patients with giant aneurysms (≥8 mm), with or without stenosis, require treatment with low-dose ASA combined with warfarin (targeting an INR of 2.0–3.0) or LMWH (if regular INR checking is difficult) **(LOE:2C)**. • KD patients with a significant risk of thrombosis should be considered for triple therapy with ASA, clopidogrel, and warfarin or LMWH **(LOE:5D)**.	8.73±0.83	92.3	H
**What is the prevention and treatment of thrombosis in patients with coronary aneurysms?**	• In KD patients with a relevant risk of thrombosis, triple therapy with ASA clopidogrel, and warfarin or LMWH should be considered in these patients **(LOE:5D)**. • Interventional cardiac catheterization or thrombolytic medication should be used to treat coronary artery thrombosis with actual or impending lumen obstruction. Low doses of ASA and low doses of heparin must be used in conjunction with thrombolytic medications, and the risk of bleeding must be carefully monitored **(LOE:1A)**. • Recombinant tissue plasminogen activator (rtPA) (alteplase) is the first-choice thrombolytic drug in children with KD complicated by coronary artery thrombosis, with a dose of (0.5 mg/kg, 10% infused over 1–2 min, and the remainder over 60 min). It is more commonly used in combination with low-dose ASA and intravenous heparin. Thorough coagulation tests monitoring every 6 to 12 h is necessary to prevent bleeding **(LOE:5D)**. • Abciximab, a glycoprotein IIb/IIIa inhibitor, may be used in cases of thrombosis with a high risk of occlusion in conjunction with low-dose ASA and intravenous heparin. Abciximab is administered intravenously (as a bolus of 0.25 mg/kg in 30 min, then followed by 0.125 g/kg/min, max: 10 g/min, for 12 h) **(LOE:5D)**.	8.61±0.89	96.2	H
**What is the treatment for patients with acute KD and complicated macrophage activation syndrome?**	• For patients with acute KD and suspected or diagnosed MAS, treatment with IVIG for KD and additional agents to treat MAS is strongly recommended **(LOE:4C)** • IVIG should be used as the first-line therapy for KD, and MAS should also be treated with the proper drugs that target cytokine storms or underlying triggers. In contrast to a primary HLH-directed treatment protocol with cytotoxic drugs, anakinra, and glucocorticoids are preferred for treatment in acute KD patients and complicated with MAS syndrome.	8.8±0.63	96.2	H
**What about vaccinations in a child with KD?**	• Since receiving any vaccine does not enhance the risk of KD recurrence, vaccinations are to be given in KD patients. **(LOE:3B)** • Inactivated vaccines, Rotavirus, oral typhoid, intranasal anti-flu, BCG vaccinations, and the yellow fever vaccine can all be given to KD patients at any time following IVIG therapy. **(LOE:4C)** • Live attenuated vaccines such as mumps, measles, rubella, and varicella should be given 10–12 months after IVIG therapy in order to avert a lowered immune response in KD patients. **(LOE:5D)** • Influenza vaccine is strongly recommended for KD patients on ASA because of the increased risk of Reye’s syndrome **(LOE:3B)** • Clopidogrel can be used in instead of ASA in KD patients who must receive the varicella or MPRV vaccines because of a potential risk of Reye’s syndrome related to the attenuated varicella-zoster virus in these vaccines. After 48 h of discontinuing ASA, V, or MPRV vaccine can be given, and ASA can be reintroduced following discontinuing clopidogrel 6 weeks after vaccination. **(LOE:5D)** • It is recommended that patients with KD on biological treatments receive all inactivated vaccines according to the regular schedule. **(LOE:4C)** • Live attenuated vaccines should be given to KD patients one month prior to starting biologic therapy; however, they are not recommended during biologic therapy. **(LOE:5D)**	8.69±0.74	96.2	H
**What is the long-term follow-up?**	Follow-up of KD patients must continue over time, especially for those who have presented coronary artery aneurysm and it depends on cardiovascular risk classes 1. **CLASS I (no abnormality of coronary arteries in the various phases of the disease)** • Cardiologic assessments (ECG, echocardiography, blood pressure monitoring), any blood chemistry investigations, and lipid profile evaluation after 12 months from the onset of the disease. 2. **Class II (transient coronary artery ectasia that disappears within 8 weeks)** • Cardiologic assessments (ECG, Echo, blood pressure monitoring), blood chemistry tests, and lipid profile evaluation at 6 and 12 months following the onset of the disease. 3. **Class III (single aneurysm of small-medium caliber between + 3 and + 7 SD in one or more arteries)** • Depending on the severity of the lesions, cardiovascular assessments (ECG, echocardiography, blood pressure monitoring, and blood chemistry testing) should be performed every 4–6 months. Cardiologic exams (ECG, echocardiography, blood pressure monitoring) should be performed annually for the first 3 years, thereafter every 3 to 5 years, if there has been a complete regression of aneurysms as shown by two successive negative controls (up to 18 years). • Assessment of myocardial perfusion every 2 years above the age of 10 (stress-ECG and/or stress echocardiogram) as well as lipid profile evaluation. • If any myocardial ischemia is detected, a coronary angiography or CT angiography should be performed. 4. **Class IV (one or more aneurysms ≥7 SD, including multiple and complex giant aneurysms without any obstruction)** • Cardiologic assessments (ECG, echocardiography, blood pressure monitoring, and blood chemistry tests) every 4 months until a stable decrease in aneurysms is confirmed by two subsequent negative controls. Cardiology evaluations and blood tests along with a stress ECG, stress echocardiography, or stress MRI with contrast to assess myocardial perfusion should be done annually. • Coronary angiography or coronary CT angiography over the first 6–12 months and after that as indicated. 5. **Class V (coronary artery obstruction at the angiography)** • Cardiologic assessments every 3 months, including ECG, echo, and maybe a Holter-ECG; annual evaluations of myocardial perfusion (stress ECG, stress echocardiogram, stress MRI with contrast). • Coronary angiography or coronary CT angiography to guide treatment decisions and total body angio-CT in the event of myocardial ischemia. • Coronary angiography or coronary CT angiography during the first 6 to 12 months, and then as needed or indicated by non-invasive investigations. **Subject to the individual patient clinical status, periodic follow-up for the long-term sequelae of the ocular complications.**	8.85±0.46	100	H

*KD* Kawasaki disease, *hpf* high-power field, *CAA* coronary artery aneurism, *ALT* alanine transaminase, *AST* aspartate transaminase, *MIS-C* multisystem inflammatory syndrome in children, *CBC* complete blood count, *DSCT* dual-source CT, *LV* left ventricle, *IVIG* intravenous immunoglobulin, *rtPA* recombinant tissue plasminogen activator, *ECG* electrocardiogram, *MAS* macrophage activation syndrome, *AASA* acetylsalicylic acid, *NSAID* nonsteroidal anti-inflammatory drug, *LMWH* low molecular weight heparin

**Fig. 1:**
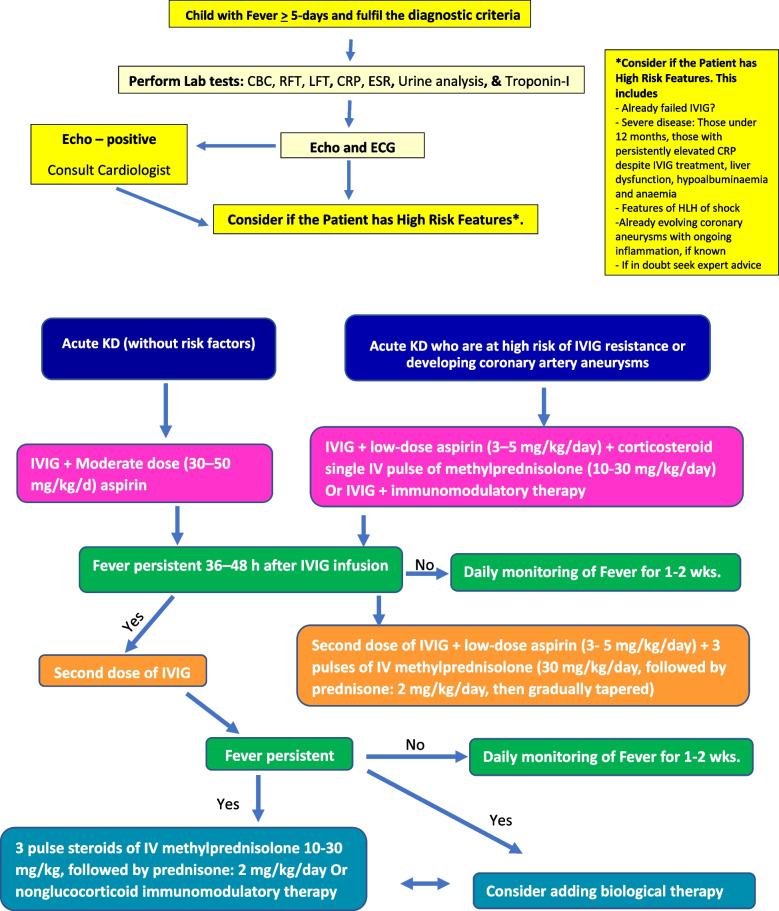
Algorithm of management of Kawasaki disease

**Fig. 2:**
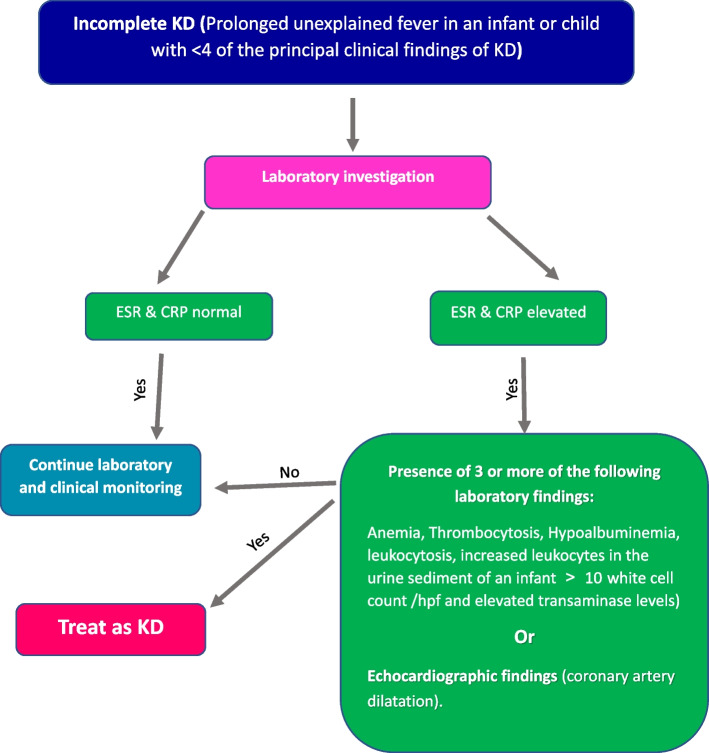
Algorithm of management of incomplete Kawasaki disease

**Fig. 3:**
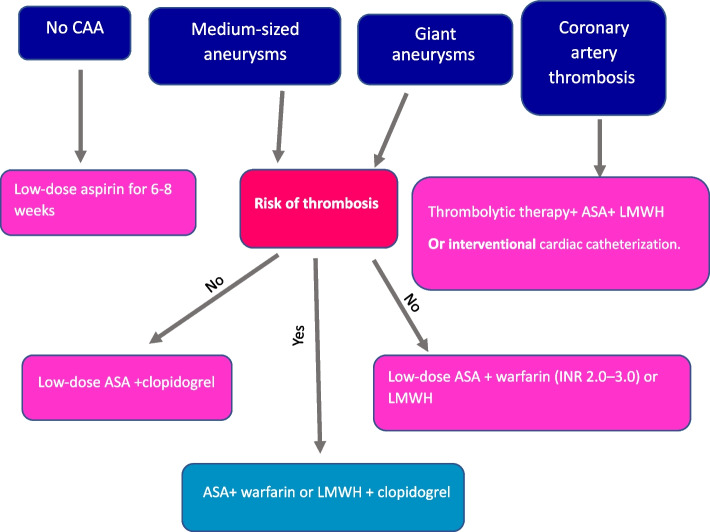
Algorithm of maintenance treatment in the patient with KD after an acute attack

Figures 1, 2, and 3 show different algorithms for KD management.

#### Recommendations for the management of children with KD

At the end of round 3, a total of seventeen recommendation items, categorized into overarching principles and 2 sections (6 recommendations are considered for diagnosis, prognosis, and monitoring of KD and 11 recommendations in the management section), were obtained.

## Discussion

The rationale behind the development of this guideline includes both the prevalence of the disease being the second most common systemic vasculitis in childhood and the fact that there are no Egyptian recommendations published for the treatment of the disease. Furthermore, over the past few years, extra evidence-based outcomes have been published on the management of KD. Therefore, such updated guideline is important as it may lead to prescribing the appropriate medical therapy, which consequently would reduce the risk of poor disease control or the development of cardiac complications.

The developed guideline provided evidence-based consensus recommendations for the diagnosis, treatment, and management of KD in children. Seventeen clinical questions were developed by the Core in the Patient/Population, Intervention, Comparison, and Outcomes (PICO) format. The systematic literature reviews were undertaken by the literature review team. The Expert Panel provided expert knowledge to develop the statements based on an informed discussion of the PICO questions and the literature review findings. In general, the development guidelines agree broadly with the treatment recommendations and guidelines published earlier, though there were some differences. Initially, this guideline provided a specific definition for the typical, atypical, and incomplete KD. Atypical KD was not included in the ACR guideline for the management of KD [15], whereas the American Heart Association guidelines [16] gave the atypical KD the same definition as incomplete KD. Several children may present with some but not all of the KD clinical features, however, may still remain at high risk of developing coronary artery aneurysms. This may cause some confusion in identifying the incomplete or atypical KD, particularly clinical manifestations that may develop sequentially, in that an incomplete or atypical case can progress into a complete case [16–20]. To predict high-risk cases, this guideline endorsed the adoption of a pragmatic strategy to draw an overall picture of disease severity based on clinical manifestations and laboratory measures, parameters that predict high-risk cases include young age (i.e., <12 months), C-reactive protein higher than 200 mg/l, albumin ≤ 2.5 g/dL, Hb at least 2 g/dL below the lower limit of normal for age, liver dysfunction: AST or ALT ≥ 2x upper limit of normal and/or direct bilirubin > 1 mg/dL, overt coronary artery aneurysms, macrophage activation syndrome (MAS) or septic shock, as well as Kobayashi score ≥ 4. This agrees with European consensus-based recommendations for the diagnosis and treatment of Kawasaki disease [21].

The recommendation presented by the *American Heart Association (*AHA) [16] was targeted mainly for the initial and long-term treatment of KD. Although the ACR recommendations [15] were designed to complement the AHA recommendations and provide extra information for rheumatologists who may be less experienced with KD, they did not mention the systemic complications linked to the acute KD’s active inflammatory process. The developed guideline has taken this extra step and provided specific recommendations for the treatment of ophthalmologic manifestation of KD, arthritis, and MAS as well as prevention and treatment of thrombosis in patients with coronary aneurysms. In agreement with the European recommendations (the SHARE initiative) [21] which included reference to immunization, this guideline provided also recommendations on vaccinations in a child with KD. In contrast neither the ACR [17], nor the Spanish [22] provided recommendations for the immunization for KD patients. This is of importance given the potential lack of effectiveness following IVIG [23, 24].

Kawasaki disease (KD) is a hybrid condition at the junction of infectious diseases, immunology, rheumatology, and cardiology [25]. The disease may be the distinctive manifestation of an immune-mediated vascular inflammation pathway in genetically susceptible children [26]. The clinical experience plays an important role in recognizing and differentiating classic KD patients from other patients who sustained other illnesses and presented with symptoms similar to the KD. In contrast to the European [21] and Spanish [22] recommendations, this guideline includes the similarities between KD and the recently identified multisystem inflammatory syndrome in children (MIS-C) associated with SARS–CoV-2 infection, infectious illnesses, sarcoidosis, and acrodynia. Though the identification of viral or bacterial agents cannot exclude KD diagnosis, it is important to consider such disorders particularly since the world is currently experiencing a pandemic caused by severe acute respiratory syndrome coronavirus 2 infection (SARS-CoV-2).

All the recommendations published in this guideline aim at facilitating improvement and uniformity of care. All the recommendations for diagnosis and treatment were accepted with a high grade of agreement. Though this guideline can serve as a resource for basic principles of management of KD, caution should be exercised in interpreting the data. As the results of future studies may necessitate amendment of the conclusions or recommendations in this guideline. In the interests of certain patients and unique situations, it might be necessary or even beneficial to depart from the guidelines. Following rules strictly may not serve as a defense against a negligence lawsuit, and following them inconsistently should not necessarily be considered negligent.

In conclusion, Kawasaki disease is the second most common systemic vasculitic disease, after IgA vasculitis, in children. The heterogeneity and complexity of KD presentation, wide-ranging differential diagnosis, and lack of a diagnostic test can be important barriers for making a prompt diagnosis. The developed evidence-based consensus recommendations for the diagnosis and management of KD represent an up-to-date document that focuses on clinical management questions which are generally posed to healthcare professionals involved in the management of KD. This guideline was developed considering experience with and availability of treatment and diagnostic options in Egypt.

## Data Availability

The data will be available upon reasonable request.

## Abbreviations

*AHA*: *American Heart Association*
ALT: Alanine transaminase
ASA: Acetylsalicylic acid
AST: Aspartate transaminase
CAA: Coronary artery aneurism
CBC: Complete blood count
DSCT: Dual-source CT
LV: Left ventricle
ECG: Electrocardiogram
hpf: High power field
IVIG: Intravenous immunoglobulin
KD: Kawasaki disease
LMWH: Low molecular weight heparin
MAS: Macrophage activation syndrome
MIS-C: Multisystem Inflammatory Syndrome in Children
NSAID: Nonsteroidal anti-inflammatory drug
PICO: Patient/Population, Intervention, Comparison, and Outcomes
RCTs: Randomized controlled trials
rtPA: Recombinant tissue plasminogen activator
SARS-CoV-2: Severe acute respiratory syndrome coronavirus 2 infection
